# Prostatic Artery Embolization (PAE) and Transurethral Resection of the Prostate (TURP) have a Differential Impact on Lower Urinary Tract Symptoms (LUTS): Retrospective Analysis of the Multicentre UK-ROPE (UK Register of Prostate Embolization) Study

**DOI:** 10.1007/s00270-021-02821-5

**Published:** 2021-04-06

**Authors:** Ganesh Vigneswaran, Drew Maclean, Mohammed Hadi, Benjamin Maher, Sachin Modi, Timothy Bryant, Mark Harris, Nigel Hacking

**Affiliations:** 1 Department of Interventional Radiology, University Hospital Southampton, Tremona Road, Southampton, SO16 6YD UK; 2 Cancer Sciences, University of Southampton, Southampton General Hospital, Tremona Road, Southampton, SO16 6YD UK; 3 Department of Urology, University Hospital Southampton, Tremona Road, Southampton, SO16 6YD UK

## Abstract

**Purpose:**

To compare the relative IPSS (International Prostate Symptom Score) improvement in storage and voiding symptoms between prostatic artery embolization (PAE) and transurethral resection of the prostate (TURP).

**Method:**

Retrospective analysis of the UK-ROPE (UK Register of Prostate Embolization) multicentre database was conducted with inclusion of all patients with full IPSS questionnaire score data. The voiding and storage subscore improvement was compared between interventions. Student’s t-test (paired and unpaired) and ANOVA (Analysis of variance) were used to identify significant differences between the groups.

**Results:**

146 patients (121 PAE, 25 TURP) were included in the analysis. Storage symptoms were more frequently the most severe symptom (‘storage’ in 75 patients vs ‘voiding’ in 17 patients). Between groups, no significant difference was seen in raw storage subscore improvement (TURP 4.9 vs PAE 4.2; *p* = 0.34) or voiding subscore improvement (TURP 8.4 vs PAE 6.7; *p* = 0.1). ANOVA demonstrated a greater proportionate reduction (relative to total IPSS) towards voiding symptoms in the TURP group (27.3% TURP vs 9.9% PAE, *p* = 0.001).

**Conclusion:**

Although both TURP and PAE improve voiding symptoms more than storage, a significantly larger proportion of total symptom reduction is due to voiding in the TURP cohort, with PAE providing a more balanced improvement between voiding and storage.

## Introduction

Prostatic artery embolization (PAE) is an effective treatment for benign prostatic obstruction (BPO) reflected by several national and international guidelines [1]. The precise role should play in the management of patients with BPO and long-term outcomes are forthcoming. It is particularly suited for younger patients looking for a non-surgical option and preservation of sexual function, or those patients with a large prostate [2]. Further indications for PAE over surgery are yet to be firmly established. This is partly because factors predictive of a good outcome are still emerging and the ideal target patient population is still taking shape [3–8].

The mechanism of action of PAE appears to be entirely different from surgery [9], and therefore it is reasonable to hypothesise it could influence symptoms in an entirely different manner. Identifying a differential symptom profile is important because it could identify patients who would be more suited to PAE over surgery. Three recent studies examined how the IPSS (International Prostate Symptom Score) breakdown changes with PAE and identified a significant reduction in both storage and voiding symptoms [10–12]. TURP (transurethral resection of the prostate) is also known to have an impact on both symptom types, but several papers have identified a reduced impact on storage symptoms [13, 14].

The UK-ROPE (UK Register of Prostate Embolization) multicentre study found no evidence of non-inferiority of PAE compared with TURP [15]. The IPSS breakdown scores for each question were recorded in the study, but these values have not been studied or published. We aimed to compare the relative IPSS improvement in storage and voiding symptoms between PAE and TURP.

## Materials and Methods

### UK-ROPE Study Population.

The UK-ROPE database was a prospective multicentre registry involving 305 patients in 17 UK urological/interventional radiology centres. In total, 216 of patients were recruited to PAE and 89 to surgery (TURP/ HoLEP- Holmium laser enucleation of the prostate). Patients were not randomised. Funding for the original registry was via support from British Society of Interventional Radiology (BSIR), British Association of Urological Surgeons (BAUS) and an Industry research grant from Cook Medical (Bloomington, Indiana, United States). The primary outcome was the global IPSS improvement at 12 months post-procedure, but data were also collected on the individual questions of the IPSS (including voiding and storage symptoms) which has not been published in the initial papers describing the study findings. Of the original cohort, a total of 254 patients had 12 month follow-up (189 PAE, 65 TURP) after removal of withdrawals, exclusions, reoperations or deaths [15].

### Inclusion and Exclusion Criteria.

All patients underwent PAE or surgery (TURP/ HoLEP) to treat lower urinary tract symptoms (LUTS) secondary to benign prostatic hyperplasia (BPH). Health research authority approval was granted by the research ethics committee, and adherence to the ethical principles of the Helsinki declaration was always maintained. Inclusion criteria: IPSS > 14 or QoL (quality of life) > 3, prostate volume > 40 ml, patients aged 50–80, eGFR (estimated glomerular filtration rate) > 45 ml min ^− 1^ m ^− 2^. Patients were excluded if they did not have the full IPSS breakdown score recorded at baseline and 12 months. Patients with long-term urinary catheters were not excluded from the study.

Baseline values collected for the registry included IPSS and individual symptom scores which make up the IPSS, QoL and International Index of Erectile Function (IIEF) via standardised questionnaire. Uroflowmetry (maximum urinary flow rate, Qmax; post-void residual volume, PVR) and serum prostatic specific antigen (PSA) were performed prior to PAE, in addition to prostate size estimation through pre-procedural CT (in PAE patients only).

The planned follow-up period for all patients was 12 months, through IPSS and IIEF questionnaires. IPSS questionnaires were also performed at 3 months post-procedure. PAE patients were imaged with a 3-month MRI scan, but this was not performed in TURP patients (Table 1)

**Table 1:** International Prostate Symptom Score Questionnaire

Symptom	Question
Frequency (Storage)	How often have you had to urinate less than every two hours?
Urgency (Storage)	How often have you found it difficult to postpone urination?
Nocturia (Storage)	How many times did you typically get up at night to urinate?
Intermittency (Voiding)	How often have you found you stopped and started again several times when you urinated?
Weak stream (Voiding)	How often have you had a weak urinary stream?
Straining (Voiding)	How often have you had to strain to start urination?
Incomplete Emptying (Voiding)	How often have you had the sensation of not emptying your bladder?

### Retrospective Statistical Analysis.

Patients were only included in this retrospective analysis if a full record of their individual IPSS question scores was recorded pre-procedure and at 12 months.

Analysis focused on ‘global IPSS reduction’- change in the total IPSS, ‘storage subscore reduction’- change in the sum of individual storage symptoms, ‘voiding subscore reduction’- change in the sum of individual voiding symptoms, ‘individual symptom reduction’- change in an individual question on the IPSS, and ‘subscore proportionate reduction’- the change in voiding/ storage symptoms relative to the patients’ global IPSS. The aim of the ‘subscore proportionate reduction’ was to identify the proportion of a patients’ IPSS improvement due to voiding symptoms and is similar to other recent studies [12]. A positive value indicates a greater reduction in voiding symptoms, whereas a negative value would indicate more of the IPSS improvement due to storage symptoms.

Baseline characteristics and post procedure IPSS values were analysed with ANOVA (analysis of variance). A significance level of 0.05 was used. For the ‘subscore proportionate reduction’ analysis, the concept was to statistically quantify the degree of symptom improvement related to storage/voiding symptoms for each intervention group. It was calculated as follows: total voiding score at baseline was divided by the total IPSS score at baseline and expressed as a percentage. Subsequently, the voiding symptom score at 12 months post procedure was divided by the total IPSS at 12 months and expressed as a percentage. Finally, the ‘voiding subscore proportionate reduction’ was calculated by subtracting the 12-month percentage from the baseline percentage. The resulting figure represents how much symptom improvement can be attributed to voiding improvement; the higher the value, the more significant the role played by voiding improvement in the overall IPSS reduction. Conversely, a negative value would indicate the symptom improvement is mostly related to a storage improvement.

Figures include data means (solid red line) with 95% confidence intervals shaded in red. The standard deviation is shaded in blue. Where raw data are shown, it has been plotted as grey circles along the Y axis. All statistical tests were performed using Matlab (MathWorks, USA).

## Results

### Baseline Characteristics.

**Fig. 1:**
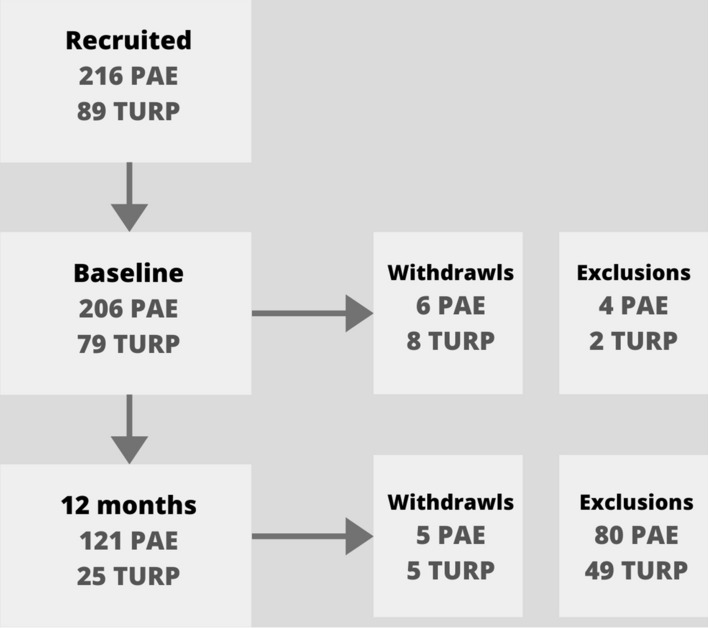
Patient inclusion/exclusion flow diagram for PAE (prostatic artery embolization) and TURP (transurethral resection of the prostate). Withdrawals were due to patient requested withdrawal. Exclusions were due to incomplete IPSS (International Prostate Symptom Score) breakdown, reoperations or deaths

Of the 216 PAE and 89 TURP patients recruited, 6 PAE and 8 TURP were withdrawn at baseline and 5 PAE and 5 TURP withdrawn within 12 months follow-up. 4 PAE and 2 TURP exclusions were made a baseline with 80 PAE and 49 TURP exclusions due to incomplete IPSS symptom breakdown data available (Fig. 1). This resulted in a total of 146 patients (121 PAE patients and 25 TURP patients) for this retrospective analysis. Baseline demographics included mean age of 66.2 years ± 6.9 [mean ± s.d], with those undergoing PAE having a significantly lower age than those undergoing TURP (PAE 65.5 vs TURP 69.4 years; *p* = 0.009, Table 2).

**Table 2:** Baseline values of our study population of PAE (prostatic artery embolization)/TURP (transurethral resection of the prostate) cohorts

	PAE (*n* = 121)	TURP (*n* = 25)	*p* value
Age (mean ± s/d)	65.5 (6.7)	69.4 (7.1)	0.009
Prostate volume (mean ± s/d)	101.9 (56.1)	N/A	N/A
PSA (prostate-specific- antigen) (mean ± s/d)	5.9 (5.1)	5.7 (7.2)	0.92
Qmax (maximum urinary flow rate) (mean ± s/d)	9.5 (9.8)	12.6 (9.0)	0.26
PVR (post-void residual volume) (mean ± s/d)	174 (153)	226 (149)	0.24
Baseline IPSS (International prostate severity score) (mean ± s/d)	21.1 (6.8)	19.9 (8.2)	0.45
QoL (quality of life) (mean ± s/d)	4.6 (1.2)	4.8 (1.0)	0.45

Prostate volume was only recorded in the PAE arm with a mean volume of 102 ml ± 56 [mean ± s.d] as part of the UK-ROPE methodology. An overall mean Qmax of 9.9 ml/s ± 9.7 was recorded across patients. When PAE and TURP groups were compared, no significant difference in baseline clinical data of PSA, Qmax, PVR, total IPSS or QoL measurement was identified. An average total IPSS of 21.1 ± 6.8 and 19.9 ± 8.2 was noted for PAE and TURP patients, respectively. The highest scoring individual symptom at baseline was nocturia (*n *= 35, 24%) followed closely by urgency (*n* = 29, 20%) (Table 3). Both symptoms are ‘storage’ symptoms. Most patients (*n* = 54, 37%) had multiple symptoms as their most severe/highest scoring, but when one symptom was most severe, this was most commonly a storage symptom (*n* = 75, 51.4%).

**Table 3:** Highest scoring/most severe individual symptom per patient

Most severe symptom	*n* = 146
Poor emptying	5
Weak stream	9
Straining	3
Intermittency	0
Frequency	11
Urgency	29
Nocturia	35
No single ‘most severe symptom’	54

**Fig. 2:**
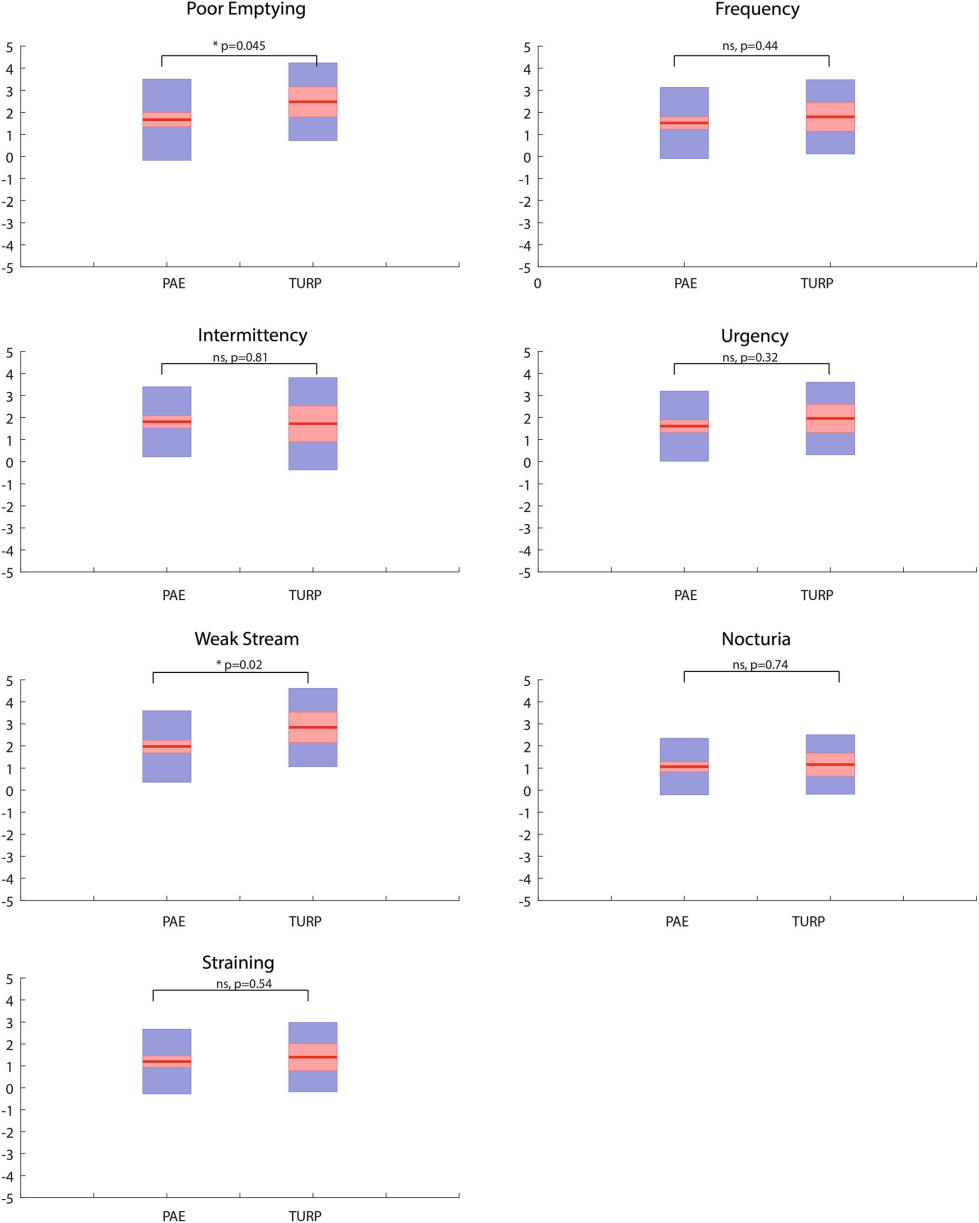
Improvement in individual IPSS (International Prostate Symptom Score) symptoms from baseline to 12 months—a comparison of PAE (prostatic artery embolization) and TURP (transurethral resection of the prostate). The mean is represented with a solid red line. 95% confidence intervals are shaded in red. The standard deviation is shaded in blue

**Fig. 3:**
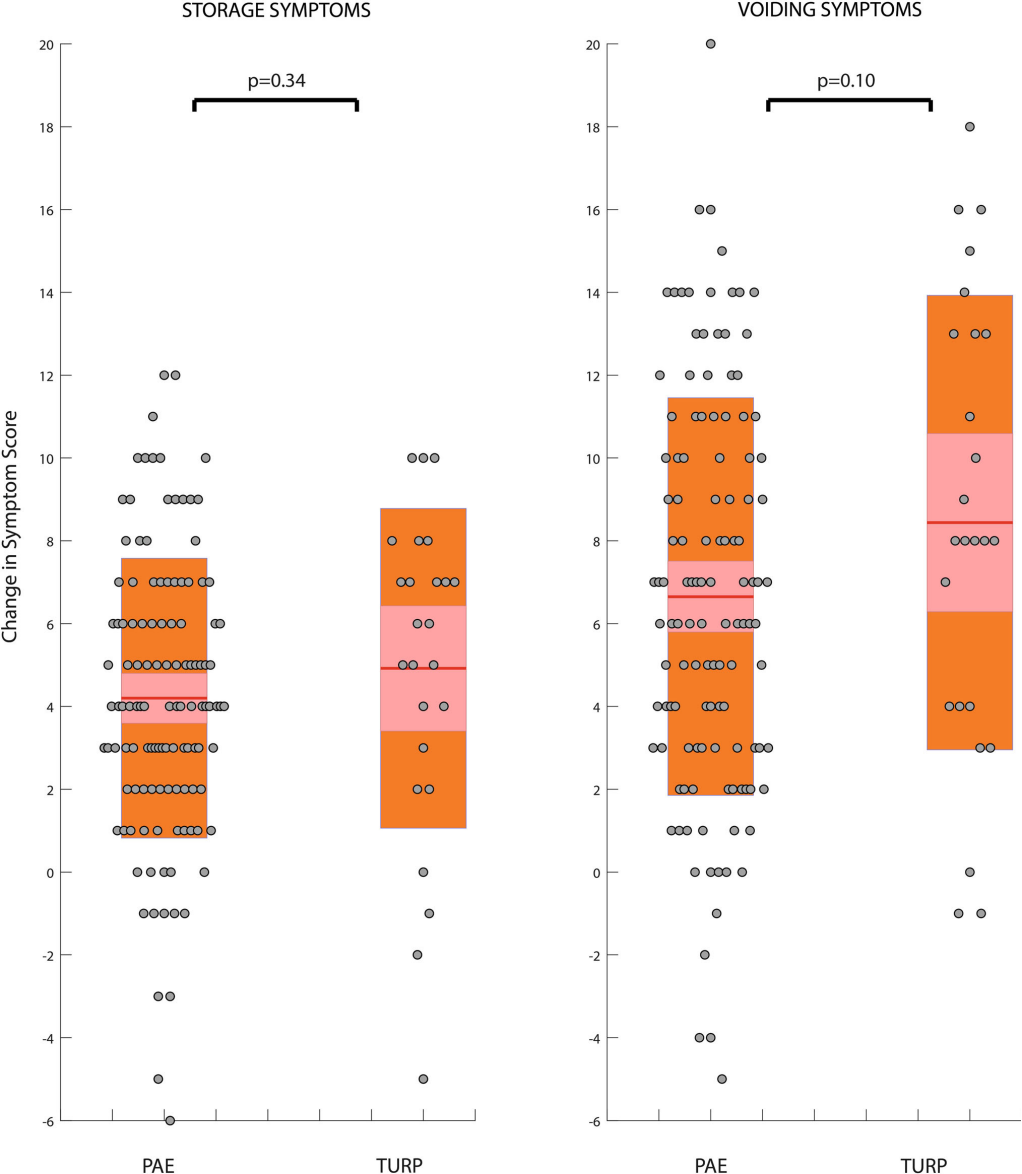
Storage and voiding subscore improvement for PAE (prostatic artery embolization) and TURP (transurethral resection of the prostate). The mean is represented with a solid red line. 95% confidence intervals are shaded in red. The standard deviation is shaded in orange. Raw data are shown as grey circles along the Y axis

We found no significant difference in overall IPSS reduction after 12 months post procedure in this limited cohort of the UK-ROPE study, although there was a trend towards a greater reduction in the TURP cohort vs PAE (13.4 vs 10.9; *p* = 0.13). Analysis of the ‘voiding’ individual questions revealed that poor emptying (TURP 2.5 vs PAE 1.7; *p* = 0.045) and weak stream (TURP 2.8 vs PAE 2.0; *p* = 0.02) were particularly and significantly improved in the TURP group over PAE (Fig. 2). TURP improved the voiding subscore to a greater extent at 12 months from baseline, although this was not significantly different compared with PAE (8.4 vs 6.7; *p* = 0.1) (Table 3, Fig. 3). PAE did not improve any symptoms to a significantly greater degree than TURP.

**Fig. 4:**
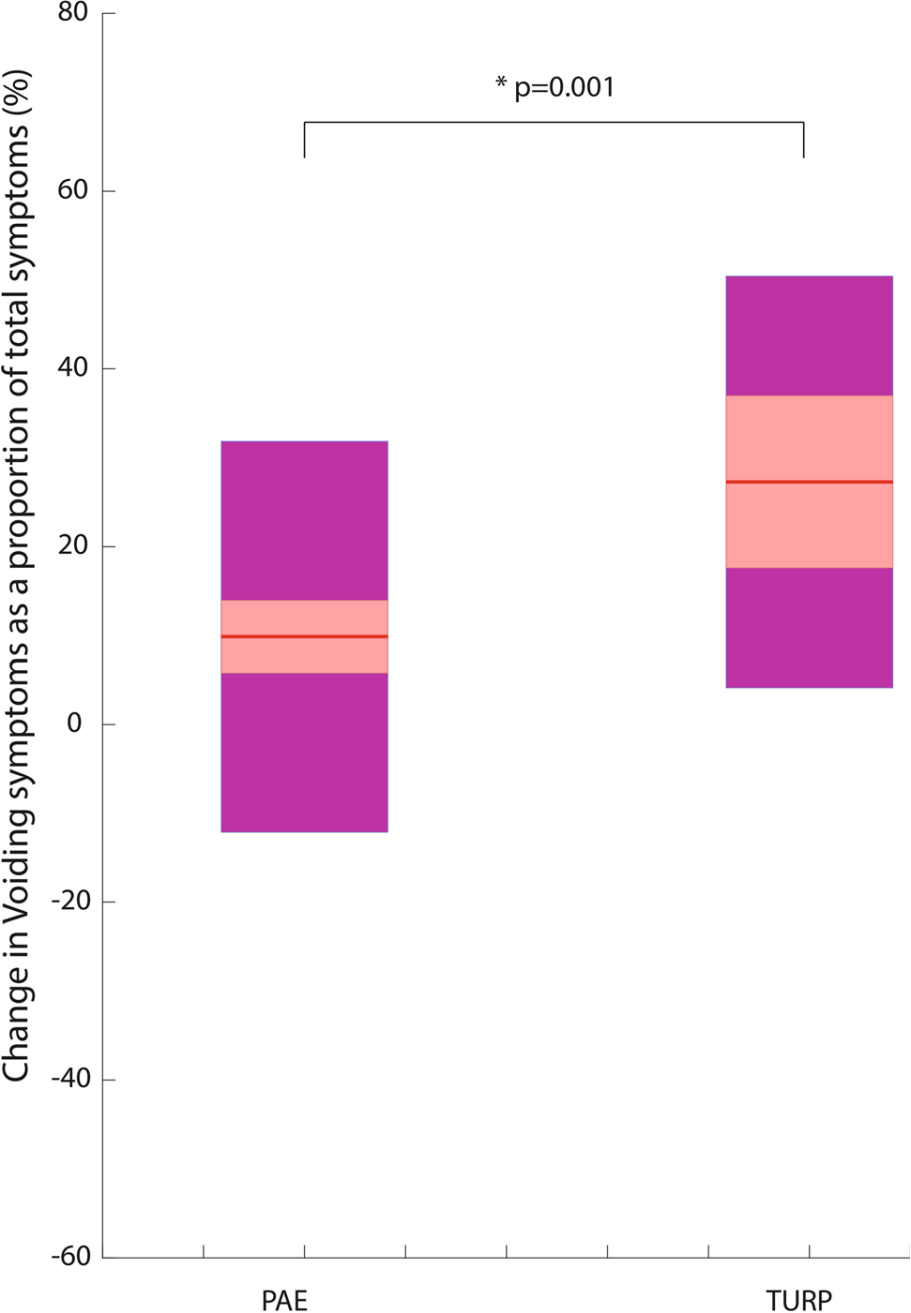
Subscore proportionate reduction—change in voiding symptoms as a proportion of total symptom improvement for PAE (prostatic artery embolization) and TURP (transurethral resection of the prostate). The mean is represented with a solid red line. 95% confidence intervals are shaded in red. The standard deviation is shaded in magenta

Improvement in the storage subscore was not significantly different between modalities (TURP 4.9 vs PAE 4.2; *p* = 0.34). Analysis of individual storage symptoms did not highlight any individual storage symptom that was significantly different between groups (Table 4). ANOVA assessment of the subscore proportionate reduction (see methods for calculation technique) showed that TURP resulted in a significantly greater proportionate change in voiding symptoms compared with PAE (27.3% TURP vs 9.9% PAE, *p* = 0.001, Fig. 4).

**Table 4:** Improvement in Individual IPSS (International Prostate Symptom Score) question score between baseline and 12 months for PAE (prostatic artery embolization) and TURP (transurethral resection of the prostate)

Voiding	PAE	TURP	*p* value
Poor emptying (mean ± s/d)	1.7 (1.8)	2.5 (1.8)	0.045
Intermittency (mean ± s/d)	1.8 (1.6)	1.7 (2.1)	0.81
Weak stream (mean ± s/d)	2.0 (1.6)	2.8 (1.8)	0.02
Straining (mean ± s/d)	1.2 (1.5)	1.4 (1.6)	0.54
Overall voiding	6.7 (4.8)	8.4 (5.5)	0.10

Storage	PAE	TURP	*p* value
Frequency	1.5 (1.6)	1.8 (1.7)	0.44
Urgency	1.6 (1.6)	2.0 (1.6)	0.32
Nocturia	1.1 (1.3)	1.2 (1.3)	0.74
Overall storage	4.2 (3.4)	4.9 (3.9)	0.34
Overall IPSS reduction	10.9 (7.4)	13.4 (8.3)	0.13

Both groups had similar baseline levels of QoL (PAE 4.6 vs TURP 4.8; *p* = 0.45). At 12 months, QoL improved in both, but it was not significantly different between treatments (PAE 2.0 vs TURP 1.6; *p* = 0.24).

## Discussion

In this retrospective subanalysis of the ROPE dataset, both TURP and PAE improved voiding symptoms to a greater degree than storage symptoms. However, the proportionate reduction values were significantly different between the two groups; TURP resulted in a significantly greater proportionate change in voiding symptoms relative to global IPSS reduction compared with PAE (27.3% TURP vs 9.9% PAE, *p* = 0.001). This suggests TURP improved voiding symptoms proportionally to a greater degree than PAE, whereas PAE provides a more balanced improvement between voiding and storage symptoms. Storage symptoms were more commonly the highest scoring IPSS symptom (‘storage’ in 75 patients vs ‘voiding’ in 17 patients, when one symptom alone was the highest scoring).

Both PAE and TURP groups experienced a significant reduction in overall raw storage and voiding subscores as would be expected. However, we found no significant difference in raw scores between these treatment modalities (although the individual voiding symptoms of ‘weak stream’ and ‘straining’ were improved greater by TURP to a significant degree). This may reflect that TURP potentially was superior at improving voiding symptoms in the UK-ROPE cohort [11] but our patient numbers were not large enough to identify a significant difference in raw scores. Indeed, several previous studies have demonstrated a greater improvement in flow rates following TURP including the UK-ROPE cohort [11].

This differential symptom response is important for two reasons. Firstly, it supports the idea that PAE may work by a different mechanism to TURP [9] (i.e., volume reduction is not the chief role of symptom improvement, consistent with only a weak association between volume reduction and symptom improvement in the UK-ROPE and other studies). Secondly, it is important because storage symptoms were most found to be a patient’s most severe (highest IPSS scoring) symptom. Parity between PAE and TURP with respect to storage symptom improvement is crucial information for patients with bothersome storage symptoms who may therefore wish to choose PAE over TURP.

The conjecture of whether these findings could be generalisable to other studies is questionable. Several more recent comparative studies (including powered randomised control trials) have demonstrated parity in the global IPSS comparing TURP to PAE [1], whereas UK-ROPE found a greater symptom response in patients undergoing surgery [15]. This difference could perhaps be due to the large number of UK centres who were early in their PAE experience, but not early in their TURP experience. It would therefore be interesting to see if similar differences in the IPSS breakdown exist in other comparative studies [1]. We, therefore, urge other comparative studies to examine their IPSS breakdown data to see if similar differences exist in other cohorts.

A significant limitation of this study is the large number of patient exclusions due to incomplete IPSS breakdown scores in the UK-ROPE study. This is because the primary outcome of the original UK-ROPE study was overall IPSS, and individual question scores were not considered essential for data submission. Therefore, our analysis was based on unbalanced PAE and TURP cohort sizes. This study was a retrospective analysis of non-randomised patients and was clearly subject to bias. Due to these factors, no change in practice should be based on this study. However, future comparative studies examining PAE and TURP should consider focusing on the storage/voiding subscore, as it may confirm the suggestion that TURP is superior to PAE in primarily resolving voiding symptoms. If found to be true, patients with particularly bothersome storage symptoms should be advised that PAE appears equivalent to TURP in this respect. Due to its safety profile with minimal side-effects, PAE may be more suited to them. Alternatively, patients with particularly bothersome voiding symptoms should be advised they are unlikely to get the same symptom benefit from PAE. This also correlates with urinary flow data, which suggests TURP is more effective at improving maximum urinary flow than PAE [1].

## Conclusion

PAE and TURP both improve voiding symptoms more than storage symptoms, but in the TURP group voiding symptom improvement makes up a larger proportion of the overall improvement compared with PAE. PAE provides a more balanced voiding/ storage improvement. Both treatments improve all individual lower urinary tract symptoms (LUTS) addressed in the questionnaire.
